# ExScalibur: A High-Performance Cloud-Enabled Suite for Whole Exome Germline and Somatic Mutation Identification

**DOI:** 10.1371/journal.pone.0135800

**Published:** 2015-08-13

**Authors:** Riyue Bao, Kyle Hernandez, Lei Huang, Wenjun Kang, Elizabeth Bartom, Kenan Onel, Samuel Volchenboum, Jorge Andrade

**Affiliations:** 1 Center for Research Informatics, The University of Chicago, Chicago, Illinois, United States of America; 2 Department of Pediatrics, The University of Chicago, Chicago, Illinois, United States of America; 3 Computation Institute, The University of Chicago, Chicago, Illinois, United States of America; Children's Medical Research Institute, AUSTRALIA

## Abstract

Whole exome sequencing has facilitated the discovery of causal genetic variants associated with human diseases at deep coverage and low cost. In particular, the detection of somatic mutations from tumor/normal pairs has provided insights into the cancer genome. Although there is an abundance of publicly-available software for the detection of germline and somatic variants, concordance is generally limited among variant callers and alignment algorithms. Successful integration of variants detected by multiple methods requires in-depth knowledge of the software, access to high-performance computing resources, and advanced programming techniques. We present ExScalibur, a set of fully automated, highly scalable and modulated pipelines for whole exome data analysis. The suite integrates multiple alignment and variant calling algorithms for the accurate detection of germline and somatic mutations with close to 99% sensitivity and specificity. ExScalibur implements streamlined execution of analytical modules, real-time monitoring of pipeline progress, robust handling of errors and intuitive documentation that allows for increased reproducibility and sharing of results and workflows. It runs on local computers, high-performance computing clusters and cloud environments. In addition, we provide a data analysis report utility to facilitate visualization of the results that offers interactive exploration of quality control files, read alignment and variant calls, assisting downstream customization of potential disease-causing mutations. ExScalibur is open-source and is also available as a public image on Amazon cloud.

## Introduction

Next Generation Sequencing (NGS) technologies are promptly becoming the most popular high-throughput strategy for drug discovery and biomedical research in the post-genome era. Whole Exome Sequencing (WES) is a powerful and cost-effective approach for the detection of single-nucleotide variants (SNVs) and small insertions/deletions (InDels) in exonic regions, which represent less than 2% of the human genome and are assumed to contain ~85% of known disease-causing variants in Mendelian disorders [1]. Analysis of the sequencing data requires in-depth bioinformatics skills and tens to thousands of computer processors for mammalian-sized genomes, which generates difficulties for researchers who may not have the expertise or the access to high-performance computing (HPC) resources. Moreover, unlike microarrays, there is no standard protocol for analysis of WES data, which also depends on the biological questions of interest. Though many tools are available, great discrepancies were reported for short-read aligners and variant callers [2–4]. Despite the rapid decline of sequencing cost, it remains challenging and time consuming to analyze large amounts of sequencing data and synthesize useful biological insights.

To address these challenges, several NGS data analysis pipelines have been published that offer different functionalities and operate on various platforms [5–11]. Most pipelines implement only one aligner and/or variant caller, lacking the facility to compare and integrate results from different algorithms. Many either do not cover the entire analysis workflow from raw sequencing data to annotated variants, or are only able to detect germline (those inherited from parents) or somatic (those gained during development) mutations. While reports are often provided, few offer a portable dynamic interface for viewing both project- and sample-level results. Moreover, setting up a pipeline usually requires complex installation and configuration, which may generate challenging tasks for most inexperienced users.

Our aim is to provide researchers the capacity to perform complex and computationally-demanding data analysis that simultaneously utilizes multiple alignment and variant detection algorithms with elastic access to resources on an as-needed basis. We present ExScalibur, a suite of highly scalable WES analysis pipelines for the detection of germline and somatic mutations, with the implementation of three aligners, six germline callers, and six somatic callers. It automates the full analysis workflow from raw sequencing reads to annotated variants and provides an interactive visualization of the results. Features include real-time progress monitoring, restarting of interrupted analyses, and seamless adaptation to different platforms. ExScalibur is an open-source project and is also available as a pre-configured environment on Amazon EC2, which greatly simplifies installation and management of complex analysis.

## Methods

### Pipeline Design

**Fig 1:**
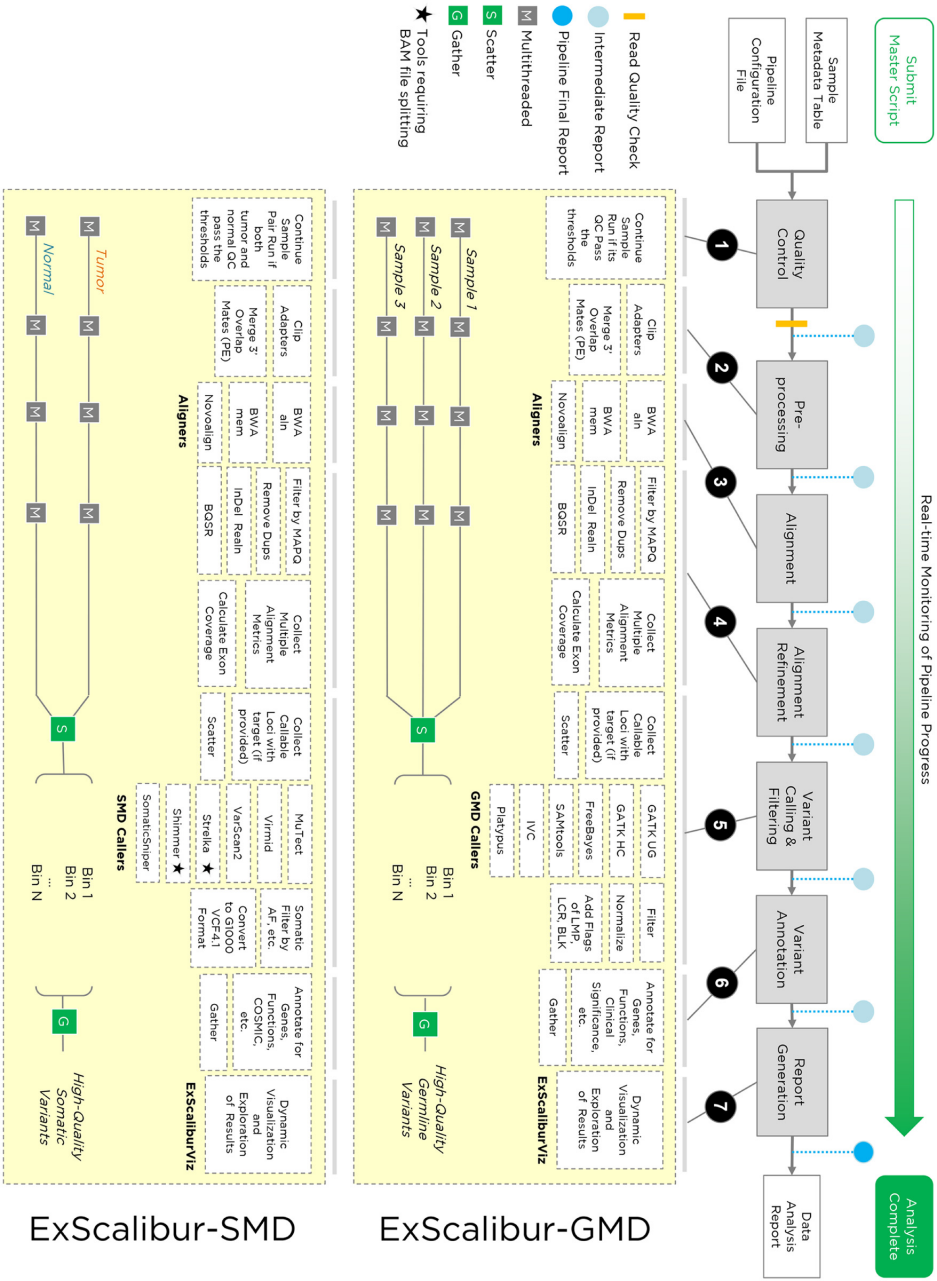
Highly modulated architecture of ExScalibur. The pipelines contain seven major analysis steps. First, the pipeline checks the quality of the sequencing reads, performs adapter trimming (for both SE and PE reads), and merges 3’ overlapping PE reads (for PE reads only). Then the reads are aligned to the reference genome, filtered, duplicates removed, and the alignment refined. The pipelines calculate exon coverage and collect callable loci from the alignment. Afterwards, the pipelines detect, filter, and annotate variants for each aligner+caller combination. Finally, the pipelines archive the results, integrate metrics and all variants sets, and generate a project data analysis report for visualization in ExScaliburViz. At the pipeline completion, a runtime report is generated to illustrate the timeline of analysis, with detailed description of the commands, inputs, outputs, and dependencies. Intermediate reports will be generated if the pipelines prematurely terminate due to software/hardware failure.

ExScalibur consists of germline (ExScalibur-GMD) and tumor/normal paired somatic mutation detection (ExScalibur-SMD) pipelines that analyze WES data generated on Illumina’s high-throughput platform. A typical analysis workflow contains seven main modules: 1) quality control (QC), 2) preprocessing, 3) alignment, 4) alignment refinement, 5) variant calling and filtering, 6) annotation, and 7) project report generation (Fig 1). Both the germline and somatic pipelines implement three short read aligners and six variant callers. Any combination of aligner and caller can be specified by the user, allowing simultaneous launching of multiple callers and direct comparison of different variant detection results (S1 and S2 Tables). At the end of analysis, the pipelines automatically collect results into the archive directory, allowing for easy downloading of essential result files.

#### Quality control

At the beginning of a pipeline run, quality of raw sequencing reads is assessed for base quality, duplication level, nucleotide composition distribution, and GC bias. Users have the option to provide the pipeline with specific metrics for these QC categories. The QC statistics are subsequently parsed to determine whether the sequencing quality passes the chosen thresholds. Samples that pass all QC criteria will be carried on to the next analysis step.

#### Preprocessing

Raw reads are processed to remove adapters (for both single- (SE) and paired-end (PE) reads by default) and to merge 3’ overlapping mates (PE reads only), for the purpose of removing the artifacts of double-counting variants located in the overlapping regions.

#### Alignment

Processed reads are mapped to the reference genome using any combination of three short-read aligners including BWA-aln [12], BWA-mem [13], and Novoalign (Novocraft Inc., Malaysia). Unmapped reads and low-quality alignments are filtered out. Alignments from technical replicates (e.g., multiple runs/lanes) are merged and read duplicates are removed.

#### Alignment refinement

The alignment is further refined by local InDel realignment and base quality score recalibration (BQSR) following the GATK Best Practices [14]. Multiple alignment summary statistics are collected and exon coverage is calculated.

#### Variant calling

ExScalibur implements parallel execution of multiple callers for increased confidence in variant detection (S1 and S2 Tables). Germline variant callers include GATK UnifiedGenotyper [15], GATK HaplotypeCaller [15], FreeBayes [16], SAMtools mpileup/bcftools [17], Isaac Variant Caller (IVC) [18] and Platypus [19]. Somatic variant callers include MuTect [20], Shimmer [21], SomaticSniper [22], Strelka [23], VarScan2 [24] and Virmid [25]. By default, variants are generated from callable exon target regions [14]. Users have the option to provide customized target regions as well. To facilitate downstream analysis, we convert and/or normalize variant calls to the 1000 Genomes Project [26] VCF4.1 format when necessary.

#### Variant filtering

Customized quality filters are applied to the raw calls to remove potential false positives (e.g., low coverage, low mapping quality, low variant quality, strong strand bias, strong read end bias, or those located within SNV clusters; S3 and S4 Tables). Somatic variants are further filtered by allele frequency (AF) in both tumor and normal samples (S4 Table). Additional flags can be added to germline variants to label those located within the ENCODE blacklist (BLK) [27] (https://sites.google.com/site/anshulkundaje/projects/blacklists), low mappability regions (LMP) [28], or low complexity regions (LCR) [13], where alignment artifacts are more likely to occur.

#### Variant annotation

Variants are annotated for gene symbol, functional changes, population frequency (e.g. the 1000 Genomes Project and the NHLBI Exome Sequence Project [29]), dbSNP ID, deleterious prediction (e.g. CADD [30] and PolyPhen2 [31]), COSMIC [32], and clinical significance (ClinVar) [33] using ANNOVAR [34]. Users may include additional annotation attributes as needed.

#### Data analysis report generation

At the completion of a pipeline run, a comprehensive data analysis report is generated, which consists of various quality statistics and variant calls. ExScalibur aggregates variants and estimates the concordance of all aligner+caller combinations using a simple multiplicative score (N_aligner_ x N_caller_). To facilitate the exploration of the results, we provide ExScaliburViz, an R Shiny [35] web application for desktop viewing (S1–S4 Figs).

### Pipeline Implementation

ExScalibur is implemented in BigDataScript (BDS), a platform-independent high-level programming language designed for pipeline development and management of large-scale data sets [36]. Utility scripts were written in Perl and Python to assist with customized pipeline functions. A project is initialized with a sample metadata table (S5 and S6 Tables) and a highly customizable pipeline configuration file. With the execution of one master script, the pipelines run from raw reads to annotated variants, and the real time progress is updated in log files. To run ExScalibur on different platforms, the only requirement from the user is to specify a handful of platform-specific parameters (S7 Table), facilitating data sharing and reproducibility in the scientific community.

#### Highly modulated architecture

ExScalibur employs a flexible dependency structure, with multiple intermediate steps that are automated by BDS (Fig 1). For example, after ExScalibur-GMD completes a run with BWA-mem and GATK HaplotypeCaller, additional aligners and callers can be added without the need to repeat already-completed upstream modules. In addition, users have the option to generate a customized analysis workflow by including specific modules from the pipelines (S8 and S9 Tables).

#### Highly scalable analysis

ExScalibur can be easily scaled to analyze tens to thousands of samples simultaneously given sufficient computing power. With a small cluster on Amazon EC2 (5 nodes; 8 cores/node; 14.6GB RAM/node), analyses of human WES data on three germline samples (80x coverage) and two tumor/normal pairs (50x coverage) involving two aligners and two variant callers finished within 12 hours and 16 hours respectively, at a cost of less than 10 US dollars per sample. To demonstrate scalability, we simulated 100 exome samples from human chromosome 22 with 1 million 2x100bp PE reads per sample, and ran the pipelines using one aligner and one caller with 32 cores on Amazon EC2. The entire analysis workflow finished within 4 hours with over 5,000 tasks successfully executed.

#### Robust handling of errors

ExScalibur captures the abnormal exit status of a task and optionally launches job resubmission through BDS [36]. If ExScalibur detects a software/hardware failure, it will gracefully handle the termination of all tasks (e.g., deleting all dependency jobs and removing incomplete files) and report detailed information of those that failed. The analysis can be restarted from the interrupted breakpoint, taking advantage of the highly modulated dependency structure.

#### Intuitive pipeline documentation

A runtime report is generated by BDS at the completion of a run, where all the commands, input and output files, and dependencies are easily accessible. In addition, the report displays an overview of the timeline of each module in an interactive graph (S1 File). A YAML format of the report is also generated that can be used for collecting runtime stats, creating custom plots and quickly retrieving commands, dependency, runtime and exit status of each task.

#### Optimized parallelization procedure

ExScalibur implements a scatter-gather design [37] for variant calling, which splits the exome callable regions into a number of even-sized bins and merges the results. This design allows for the submission of hundreds to thousands of jobs on HPC clusters and cloud infrastructures, dramatically reducing the analysis time.

#### Availability and resources

ExScalibur is available under open-source license at http://exscalibur.cri.uchicago.edu. The website hosts documentation and tutorials and provides access to an Amazon’s Elastic Compute Cloud (EC2) image with pre-installed pipeline scripts and tools. With minimal installation requirements, users may instantiate the provided image with as many resources as needed. Nodes may be added or removed on the fly and ExScalibur can immediately make use of the available hardware. The cloud image is built on StarCluster [38] running Ubuntu operating system, allowing fast and easy provision of a cluster environment in the cloud.

## Results

To evaluate the pipeline performance, we ran GMD and SMD analyses on simulated/benchmark data and 30 acute myeloid leukemia (AML) tumor/normal pairs [39]. In addition, we illustrated discrepancies between the two commonly-used somatic mutation detection approaches by using the GMD-derived subtraction method to identify somatic calls via contrasting genotypes of paired tumor/normal samples and comparing the results with those directly detected by somatic callers in the SMD pipeline.

### ExScalibur-GMD Evaluation

Raw PE reads were assessed by FastQC [40] (*see* S15 Table for all tool versions) for quality, preprocessed by SeqPrep [41], and aligned to human reference genome (hg19) using BWA-mem and Novoalign. Alignments were filtered to remove duplicates, unmapped reads, and reads with mapping quality (MAPQ) less than 30. GATK was used to realign InDel regions and recalibrate base quality score. Variants were identified using four callers (GATK HaplotypeCaller, FreeBayes, SAMtools mpileup/bcftools and IVC), filtered for confident calls, normalized (vcflib [42] and vt normalize [43]), and annotated using ANNOVAR. Unless otherwise noted, variants labeled with “HQ” (high quality) refer to those that passed all filters.

For evaluation, we focused on SNVs and small InDels, which composed the majority of the variant calls. Variants generated by every aligner+caller combination (“Observed”) were compared to a validation dataset (“Expected”). Sites detected in both the observed and expected sets were considered true positives (TPs), while sites detected as variant in the observed set but as invariant in the expected set were considered false positives (FPs). True negatives (TNs) refer to sites detected as invariant in both sets, while false negatives (FNs) refer to sites detected as invariant in the observed set but as variant in the expected set. To make the results comparable, we focused on candidate loci covered by at least six reads across all combinations.

#### NIST-GIAB benchmark data

The NIST-GIAB benchmark data were generated by the Genome in a Bottle Consortium that provides a list of high-confidence variant calls from the genome of NA12878 [44]. In this study, we identified variants from a trio including NA12878 (SRA accession ID SRX079575), NA12891 (SRX079576) and NA12892 (SRX079577) using ExScalibur-GMD pipeline with different tools.

We retrieved NA12878 variants from the multi-sample variant detection results and compared to the NIST-GIAB gold standard dataset for evaluation. We removed off-target calls and variants located within genome regions where no confident calls could be made [44]. Variants of low quality or called as multiallelic were also excluded. A total of 15,914,394 loci were included for evaluation. We retrieved variants detected by at least two aligners and two callers (S10 Table, *2aligners+2callers*). We detected the highest sensitivity in the “2aligner+2caller” list across all combinations for the detection of SNVs (99.03%). Compared to single combinations that detected over 11,000 true SNVs (e.g. BWA-mem+GATKHaplotypeCaller), this approach greatly reduced the number of false positives by recruiting multiple callers (113 versus 54). Close to 90% sensitivity was observed for the InDels, which is among the best-performing combinations but lower than that of GATKHaplotypeCaller, possibly due to large differences in the performance of the other three callers. Considering both sensitivity and precision, our results suggested that GATK HaplotypeCaller showed the best performance among the four callers.

#### AML data

We obtained WES data of 30 AML tumor/normal pairs from the TCGA portal (released December 2014). We included only the normal samples for ExScalibur-GMD evaluation. We used Affymetrix human SNP array 6.0 genotype calls as the validation set, which were processed by Washington University at St. Louis and the Broad Institute’s TCGA groups. We retrieved concordant calls detected by both groups, lifted genomic coordinates from hg18 to hg19, filtered for array sites covered by at least six reads in the AML exome data, and retrieved candidate loci consistent across all combinations within each sample. On average, 38,970 ± 14,800 loci were included in the evaluation. We caution that this analysis was restricted to a limited set of loci targeted by the SNV array, which tend to include only the common SNVs from public databases.

We detected higher than 99% precision in all combinations when averaging across all samples (Table 1). A larger discrepancy in sensitivity was observed between single aligner+caller combinations, with BWA-mem and GATK HaplotypeCaller having the best performance. Of note, variants detected by at least two aligners and two callers showed the highest sensitivity (98.23% ± 0.99%) with little tradeoff in precision (99.72% ± 0.56%) (Table 1, *2aligners+2callers*).

**Table 1:** Evaluation of GMD germline SNV detection in the AML dataset.

Variant Set	TP	FP	TN	FN	Sensitivity (SD) %	Specificity (SD) %	Precision (SD) %
BWA-mem+GATKHaplotypeCaller	15,856	33	22,564	516	97.17 (1.24)	99.86 (0.34)	99.80 (0.48)
Novoalign+GATKHaplotypeCaller	15,833	33	22,564	538	97.03 (1.24)	99.86 (0.34)	99.80 (0.48)
BWA-mem+FreeBayes	14,982	94	22,503	1,390	91.66 (0.90)	99.54 (0.42)	99.31 (0.62)
Novoalign+FreeBayes	15,009	80	22,517	1,363	91.80 (0.87)	99.62 (0.35)	99.43 (0.52)
BWA-mem+IsaacVariantCaller	11,560	11	22,586	4,812	73.53 (8.30)	99.95 (0.05)	99.90 (0.11)
Novoalign+IsaacVariantCaller	11,288	11	22,586	5,083	71.77 (8.09)	99.95 (0.05)	99.90 (0.10)
BWA-mem+SAMtools	15,153	60	22,537	1,219	91.82 (2.19)	99.75 (0.50)	99.62 (0.73)
Novoalign+SAMtools	14,210	57	22,540	2,161	85.26 (4.21)	99.76 (0.45)	99.62 (0.71)
*2aligners+2callers*	16,057	47	22,550	315	98.23 (0.99)	99.80 (0.40)	99.72 (0.56)

Counts and percentages are shown as the average across 30 AML normal samples. SD: Standard Deviation.

### ExScalibur-SMD Evaluation

Reads were processed, aligned to the hg19 assembly using BWA-mem and Novoalign, and refined as described above. Somatic variants were called using six somatic callers (MuTect, Shimmer, SomaticSniper, Strelka, VarScan2, and Virmid), followed by caller-specific filtering to remove ambiguous and low-confidence calls. After filtering, we restricted our evaluation to include only somatic SNVs that were heterozygous in the tumor sample, homozygous reference in the matched normal sample, and had at least 8x coverage.

#### Simulation data

We implemented the virtual-tumor benchmarking approach [20] to generate one dataset for the estimation of specificity and another for sensitivity (Datasets 1 and 2, respectively). Briefly, Dataset 1 was generated by randomly assigning WES reads from NA12891 to a virtual tumor/normal pair. Any somatic variants detected in the virtual sample pair were considered as false positives and used to estimate specificity (defined as 1—FP/total number of exome sites). Dataset 2 was generated using alignments from NA12891 and a second individual, NA12878. First, we retrieved high-confidence variants from the 1000 Genomes database where NA12878 was a homozygous reference and NA12891 was a heterozygous reference. Then, we simulated a virtual NA12878-tumor sample by substituting NA12891 alleles into NA12878 at a frequency of 0.8 and a minimum coverage of 20x. Any somatic variants detected in this NA12878-tumor/NA12878 pair were considered as true positives and used to estimate sensitivity (defined as TP/total number of substituted loci). To make the results comparable within each dataset, we focused on candidate loci available across all combinations.

We detected close to 90% sensitivity for all aligners when averaging across all callers (Table 2). A larger variation was observed between SMD callers, with Shimmer and Strelka having the lowest sensitivity and SomaticSniper and VarScan2 having the greatest. Specificity was above 99% across all aligner+caller combinations. However, VarScan2 and SomaticSniper detected a relatively greater number of false positives (S11 Table). After applying the default filters implemented in ExScalibur-SMD, the number of false positives detected by VarScan2 dropped down to a similar level as the other somatic callers, while the discrepancy in SomaticSniper persisted (Table 2). Of note, the combination of at least two aligners and two somatic callers produced the highest sensitivity with no tradeoff in specificity (Table 2, *2aligners+2callers*).

**Table 2:** Evaluation of SMD somatic SNV detection in the simulation datasets.

Variant Set	Dataset 1				Dataset 2			
	TP	FN	Sensitivity %	FNR	FP	TN	Specificity %	FPR
BWA-mem+MuTect	690	52	92.99	7.01E-02	16	47,301,677	99.99997	3.38E-07
Novoalign+MuTect	684	58	92.18	7.82E-02	23	47,301,670	99.99995	4.86E-07
BWA-mem+Shimmer	550	192	74.12	2.59E-01	0	47,301,693	100.00000	0.00
Novoalign+Shimmer	536	206	72.24	2.78E-01	0	47,301,693	100.00000	0.00
BWA-mem+SomaticSniper	707	35	95.28	4.72E-02	110	47,301,583	99.99977	2.33E-06
Novoalign+SomaticSniper	697	45	93.94	6.06E-02	109	47,301,584	99.99977	2.30E-06
BWA-mem+Strelka	597	145	80.46	1.95E-01	16	47,301,677	99.99997	3.38E-07
Novoalign+Strelka	596	146	80.32	1.97E-01	19	47,301,674	99.99996	4.02E-07
BWA-mem+VarScan2	708	34	95.42	4.58E-02	27	47,301,666	99.99994	5.71E-07
Novoalign+VarScan2	705	37	95.01	4.99E-02	25	47,301,668	99.99995	5.29E-07
BWA-mem+Virmid	678	64	91.37	8.63E-02	0	47,301,693	100.00000	0.00
Novoalign+Virmid	690	52	92.99	7.01E-02	4	47,301,689	99.99999	8.46E-08
*2aligners+2callers*	713	29	96.09	3.91E-02	1	47,301,692	100.00000	0.00

Results are shown for high-quality variants that passed all quality filters. Additional precision digits were kept for Specificity to infer small differences.

#### AML data

We detected somatic variants in the AML WES data using ExScalibur-SMD and compared results with validated somatic mutations generated by the TCGA group, which were identified by SomaticSniper and validated by hybridization arrays (validated somatic mutations; VSMs). We lifted genomic coordinates from hg18 to hg19. Because the use of the VSMs in our evaluation is limited to a handful of loci included in the validation panel, any somatic variants detected by ExScalibur-SMD but are not present on the panel cannot be evaluated. With this caveat in mind, we calculated two evaluation metrics for each sample: (1) Recovery rate, defined as the ratio of the number of VSMs detected in our results over the total number of VSMs; (2) Novel call rate, defined as the ratio of the number of VSMs detected in our results over the total number of somatic SNVs detected.

We observed large discrepancies in the recovery rate between aligner+caller combinations, with an average of 35% to 83% of the VSMs detected before filtering (S12 Table, VSM Recovery Rate). Of note, applying the somatic variant filters dramatically reduced the number of false positives but with a tradeoff in the recovery rate. In particular, SomaticSniper showed the most drastic drop in VSM recovery rate (from 44% to 9%) after filtering, mostly due to low genotype quality in the tumor sample. The combination of two aligners and two callers produced high recovery rates similar to VarScan2 but resulted in better performance than all other single aligner+caller combinations (S12 Table, *2aligner+2caller*). Interestingly, the majority of somatic variants detected by ExScalibur-SMD did not overlap with the VSMs, suggesting that the use of multiple callers may increase the sensitivity of somatic mutation detection.

### Comparison of somatic calls between SMD and GMD pipelines

Germline variants are usually associated with an expected ploidy-dependent allele frequency. In a diploid genome, this frequency is expected to be close to 0% for homozygous reference alleles, 50% for heterozygous alleles, and 100% for homozygous alternative alleles. In contrast, somatic variants often have an unexpected spectrum of tumor allele frequencies and ploidy changes. Moreover, reliable detection of somatic mutations is often compromised by contamination from the normal tissue. To address these issues, modern SMD software requires paired tumor/normal samples and implements complex statistical models to handle unexpected frequencies correcting for contamination rate. An alternate approach involves the detection of variants in tumor and normal samples separately and then contrasting the tumor and normal genotypes (GMD-derived subtraction approach). In this case, somatic variants were identified as sites that carry homozygous or heterozygous alternative alleles in the tumor sample but carry homozygous reference in the matched normal sample.

To compare the two somatic mutation detection approaches (the paired tumor/normal SMD vs. GMD-derived subtraction), we analyzed the AML data using both pipelines. For SMD, the approaches were described in the previous section. For GMD, we retrieved SNVs from three callers (GATK HaplotypeCaller, FreeBayes, and SAMtools) and filtered for loci that were heterozygous genotype in tumor sample (AF > 0.20) and homozygous reference in matched normal (AF < 0.05). On average, an over 80% recovery rate was observed for GATK HaplotypeCaller and SAMtools (S13 Table). Of note, a higher recovery rate was observed (92%) in variants concordantly detected by at least 2 aligners and 2 callers, with a reduction of 90% in the number of false positivies.

Overall, the GMD-subtraction method detected more than double the number of somatic mutations compared to the SMD pipeline (S14 Table). Of these, 29.03% of the somatic mutations detected by SMD overlapped with 8.32% of the somatic mutations detected by GMD-subtraction. To further investigate the large discrepancy between GMD and SMD somatic calls, we randomly selected 50 variants detected by each pipeline and visually inspected them in Integrative Genomics Viewer (IGV) [45, 46]. Our manual inspection suggested that many of the differences could be explained by discrepancies in allele frequencies, perhaps due to intrinsic differences in the variant caller algorithms between SMD and GMD. Though the GMD-subtraction method showed a higher recovery rate, we recommend using SMD for somatic variant calling due to its low FP rate and high sensitivity.

## Discussion

We introduce ExScalibur, a set of highly scalable and configurable WES pipelines. The pipelines cover the complete workflow from raw reads to variant calling and annotation, allowing accurate detection of germline and somatic variants in the human genome. ExScalibur executes the requested analysis steps, allows for fine control over software parameters (with carefully chosen default parameters), manages data across all processes, and distributes computationally expensive tasks across HPC nodes. It is available for implementation across platforms, facilitating large-scale data analysis in individual laboratories as well as institutions that process samples routinely. We also provide a ready-to-use virtual image that can be easily deployed on Amazon EC2, allowing execution of complex sequencing analyses for researchers who may not have access to a HPC environment. In our experience, ExScalibur is the first WES analysis suite implemented in the BDS language, which is equipped with unique features to manage pipeline execution and robustness for the complex analysis of big data.

Our evaluation suggests that the combination of multiple aligners and callers often results in more confident variant detection in both GMD and SMD pipelines. While low concordance was observed between somatic variant callers, we recommend using more than one caller and retrieving concordant calls detected by at least two or more somatic callers for increased sensitivity and confidence.

ExScalibur is a set of open-source pipelines that assist researchers in quickly gaining biological insights into genomic aberrations identified through exome sequencing. We believe it will be highly useful to those who do not have access to large-scale hardware resources or necessary expertise to run the analyses. More importantly, our suite of tools will provide a new framework to implement and compare different aligners and variant callers. ExScalibur is under active development and maintained for long-term use. The pipelines are under heavy use in a biomedical research environment and have successfully identified causal mutations in rare Mendelian diseases and cancer.

## Supporting Information

S1 File Pipeline runtime report of ExScalibur pipelines on sample data. Horizontal bars represent the progress of each module. Text on/next to each bar indicates sample/read group and software information. Runtime is shown as x-axis at the bottom of the panel. Task information and system settings not shown.
(HTML)

S1 Fig Project data analysis report automatically generated by ExScaliburViz. (TIF)

S2 Fig Project data analysis report automatically generated by ExScaliburViz. (TIF)

S3 Fig Project data analysis report automatically generated by ExScaliburViz. (TIF)

S4 Fig Project data analysis report automatically generated by ExScaliburViz. (TIF)

S1 Table Aligners and variant callers and their default parameters in ExScalibur-GMD pipeline. (XLSX)

S2 Table Aligners and variant callers and their default parameters in ExScalibur-SMD pipeline. (XLSX)

S3 Table Default variant call filters in ExScalibur-GMD pipeline. (XLSX)

S4 Table Default variant call filters in ExScalibur-SMD pipeline. (XLSX)

S5 Table Description of metadata table schema in ExScalibur-GMD pipeline. (XLSX)

S6 Table Description of metadata table schema in ExScalibur-SMD pipeline. (XLSX)

S7 Table Description of command-line parameters in ExScalibur-GMD pipeline. (XLSX)

S8 Table Description of pipeline flags in ExScalibur-GMD pipeline. (XLSX)

S9 Table Description of command-line parameters in ExScalibur-SMD pipeline. (XLSX)

S10 Table Evaluation of GMD germline variant detection in the benchmark dataset. (XLSX)

S11 Table Evaluation of SMD somatic SNV detection in the simulation datasets. All variants (before filtering) were included.
(XLSX)

S12 Table Evaluation of SMD SNV detection in the AML dataset. Values represent averages and standard deviations across all 30 TCGA AML tumor/normal pairs. HQ: high quality. VSM: validated somatic mutations. See context for detail.
(XLSX)

S13 Table Evaluation of GMD somatic SNV detection in the AML dataset. Values represent averages and standard deviations across 30 AML tumor/normal pairs.
(XLSX)

S14 Table Comparison of somatic SNV detection between SMD in the AML dataset. Values represent averages and standard deviations across in 30 AML tumor/normal pairs. HQ: high-quality variants.
(XLSX)

S15 Table Tools used in ExScalibur pipeline evaluation. (XLSX)

S16 Table List of AML sample IDs in the TCGA database. (XLSX)

## References

[1] Botstein D, Risch N. Discovering genotypes underlying human phenotypes: past successes for mendelian disease, future approaches for complex disease. Nat Genet. 2003;33 Suppl:228–37. 10.1038/ng1090 . 12610532

[2] O'Rawe J, Jiang T, Sun G, Wu Y, Wang W, Hu J, et al Low concordance of multiple variant-calling pipelines: practical implications for exome and genome sequencing. Genome medicine. 2013;5(3):28 10.1186/gm432 23537139 PMC3706896

[3] Kim SY, Speed TP. Comparing somatic mutation-callers: beyond Venn diagrams. BMC bioinformatics. 2013;14:189 10.1186/1471-2105-14-189 23758877 PMC3702398

[4] Roberts ND, Kortschak RD, Parker WT, Schreiber AW, Branford S, Scott HS, et al A comparative analysis of algorithms for somatic SNV detection in cancer. Bioinformatics. 2013;29(18):2223–30. 10.1093/bioinformatics/btt375 23842810 PMC3753564

[5] Li J, Doyle MA, Saeed I, Wong SQ, Mar V, Goode DL, et al Bioinformatics pipelines for targeted resequencing and whole-exome sequencing of human and mouse genomes: a virtual appliance approach for instant deployment. PloS one. 2014;9(4):e95217 10.1371/journal.pone.0095217 24752294 PMC3994043

[6] Fischer M, Snajder R, Pabinger S, Dander A, Schossig A, Zschocke J, et al SIMPLEX: cloud-enabled pipeline for the comprehensive analysis of exome sequencing data. PloS one. 2012;7(8):e41948 10.1371/journal.pone.0041948 22870267 PMC3411592

[7] Pirooznia M, Kramer M, Parla J, Goes FS, Potash JB, McCombie WR, et al Validation and assessment of variant calling pipelines for next-generation sequencing. Human genomics. 2014;8:14 10.1186/1479-7364-8-14 25078893 PMC4129436

[8] Challis D, Yu J, Evani US, Jackson AR, Paithankar S, Coarfa C, et al An integrative variant analysis suite for whole exome next-generation sequencing data. BMC bioinformatics. 2012;13:8 10.1186/1471-2105-13-8 22239737 PMC3292476

[9] Mutarelli M, Marwah V, Rispoli R, Carrella D, Dharmalingam G, Oliva G, et al A community-based resource for automatic exome variant-calling and annotation in Mendelian disorders. BMC genomics. 2014;15 Suppl 3:S5 10.1186/1471-2164-15-S3-S5 25078076 PMC4083405

[10] D'Antonio M, D'Onorio De Meo P, Paoletti D, Elmi B, Pallocca M, Sanna N, et al WEP: a high-performance analysis pipeline for whole-exome data. BMC bioinformatics. 2013;14 Suppl 7:S11 10.1186/1471-2105-14-S7-S11 23815231 PMC3633005

[11] Reid JG, Carroll A, Veeraraghavan N, Dahdouli M, Sundquist A, English A, et al Launching genomics into the cloud: deployment of Mercury, a next generation sequence analysis pipeline. BMC bioinformatics. 2014;15:30 10.1186/1471-2105-15-30 24475911 PMC3922167

[12] Li H, Durbin R. Fast and accurate short read alignment with Burrows-Wheeler transform. Bioinformatics. 2009;25(14):1754–60. 10.1093/bioinformatics/btp324 19451168 PMC2705234

[13] Li H. Towards Better Understanding of Artifacts in Variant Calling from High-Coverage Samples. Bioinformatics. 2014;30(20):2843–51. 10.1093/bioinformatics/btu356 24974202 PMC4271055

[14] Van der Auwera G, Carneiro M, Hartl C, Poplin R, del Angel G, Levy-Moonshine A, et al From FastQ Data to High-Confidence Variant Calls: The Genome Analysis Toolkit Best Practices Pipeline. Current Protocols in Bioinformatics. 2013;43:11.0.1–.0.33. 25431634 10.1002/0471250953.bi1110s43 PMC4243306

[15] DePristo MA, Banks E, Poplin R, Garimella KV, Maguire JR, Hartl C, et al A framework for variation discovery and genotyping using next-generation DNA sequencing data. Nat Genet. 2011;43(5):491–8. 10.1038/Ng.806 WOS:000289972600023. 21478889 PMC3083463

[16] Garrison E, Marth G. Haplotype-based variant detection from short-read sequencing. arXiv:12073907 [q-bioGN]. 2012.

[17] Li H, Handsaker B, Wysoker A, Fennell T, Ruan J, Homer N, et al The Sequence Alignment/Map format and SAMtools. Bioinformatics. 2009;25(16):2078–9. 10.1093/bioinformatics/btp352 19505943 PMC2723002

[18] Raczy C, Petrovski R, Saunders CT, Chorny I, Kruglyak S, Margulies EH, et al Isaac: ultra-fast whole-genome secondary analysis on Illumina sequencing platforms. Bioinformatics. 2013;29(16):2041–3. 10.1093/bioinformatics/btt314 . 23736529

[19] Rimmer A, Phan H, Mathieson I, Iqbal Z, Twigg SR, Consortium WGS, et al Integrating mapping-, assembly- and haplotype-based approaches for calling variants in clinical sequencing applications. Nat Genet. 2014;46(8):912–8. 10.1038/ng.3036 . 25017105 PMC4753679

[20] Cibulskis K, Lawrence MS, Carter SL, Sivachenko A, Jaffe D, Sougnez C, et al Sensitive detection of somatic point mutations in impure and heterogeneous cancer samples. Nature biotechnology. 2013;31(3):213–9. 10.1038/nbt.2514 23396013 PMC3833702

[21] Hansen NF, Gartner JJ, Mei L, Samuels Y, Mullikin JC. Shimmer: detection of genetic alterations in tumors using next-generation sequence data. Bioinformatics. 2013;29(12):1498–503. 10.1093/bioinformatics/btt183 23620360 PMC3673219

[22] Larson DE, Harris CC, Chen K, Koboldt DC, Abbott TE, Dooling DJ, et al SomaticSniper: identification of somatic point mutations in whole genome sequencing data. Bioinformatics. 2012;28(3):311–7. 10.1093/bioinformatics/btr665 22155872 PMC3268238

[23] Saunders CT, Wong WS, Swamy S, Becq J, Murray LJ, Cheetham RK. Strelka: accurate somatic small-variant calling from sequenced tumor-normal sample pairs. Bioinformatics. 2012;28(14):1811–7. 10.1093/bioinformatics/bts271 . 22581179

[24] Koboldt DC, Zhang Q, Larson DE, Shen D, McLellan MD, Lin L, et al VarScan 2: somatic mutation and copy number alteration discovery in cancer by exome sequencing. Genome research. 2012;22(3):568–76. 10.1101/gr.129684.111 22300766 PMC3290792

[25] Kim S, Jeong K, Bhutani K, Lee J, Patel A, Scott E, et al Virmid: accurate detection of somatic mutations with sample impurity inference. Genome biology. 2013;14(8):R90 10.1186/gb-2013-14-8-r90 23987214 PMC4054681

[26] The 1000 Genomes Project Consortium. An integrated map of genetic variation from 1,092 human genomes. Nature. 2012;491(7422):56–65. 10.1038/nature11632 23128226 PMC3498066

[27] Encode Project Consortium. An integrated encyclopedia of DNA elements in the human genome. Nature. 2012;489(7414):57–74. 10.1038/nature11247 22955616 PMC3439153

[28] Derrien T, Estelle J, Marco Sola S, Knowles DG, Raineri E, Guigo R, et al Fast computation and applications of genome mappability. PloS one. 2012;7(1):e30377 10.1371/journal.pone.0030377 22276185 PMC3261895

[29] Exome Variant Server, NHLBI GO Exome Sequencing Project (ESP), Seattle, WA (URL: http://evs.gs.washington.edu/EVS/), accessed January, 2013.

[30] Kircher M, Witten DM, Jain P, O'Roak BJ, Cooper GM, Shendure J. A general framework for estimating the relative pathogenicity of human genetic variants. Nat Genet. 2014;46(3):310–5. 10.1038/ng.2892 24487276 PMC3992975

[31] Adzhubei IA, Schmidt S, Peshkin L, Ramensky VE, Gerasimova A, Bork P, et al A method and server for predicting damaging missense mutations. Nature methods. 2010;7(4):248–9. 10.1038/nmeth0410-248 20354512 PMC2855889

[32] Forbes SA, Beare D, Gunasekaran P, Leung K, Bindal N, Boutselakis H, et al COSMIC: exploring the world's knowledge of somatic mutations in human cancer. Nucleic acids research. 2014 10.1093/nar/gku1075 . 25355519 PMC4383913

[33] Landrum MJ, Lee JM, Riley GR, Jang W, Rubinstein WS, Church DM, et al ClinVar: public archive of relationships among sequence variation and human phenotype. Nucleic acids research. 2014;42(Database issue):D980–5. 10.1093/nar/gkt1113 24234437 PMC3965032

[34] Wang K, Li M, Hakonarson H. ANNOVAR: functional annotation of genetic variants from high-throughput sequencing data. Nucleic acids research. 2010;38(16):e164 10.1093/nar/gkq603 20601685 PMC2938201

[35] RStudio_Inc. shiny: Easy web applications in R. URL: http://shinyrstudiocom. 2014.

[36] Cingolani P, Sladek R, Blanchette M. BigDataScript: a scripting language for data pipelines. Bioinformatics. 2015;31(1):10–6. 10.1093/bioinformatics/btu595 25189778 PMC4271142

[37] VdAuwera G. A primer on parallelism with the GATK. GATK documentation URL: http://googl/ia2l6I. 2013.

[38] Riley J. StarCluster website: http://star.mit.edu/cluster.

[39] The Cancer Genome Atlas Research Network. Genomic and epigenomic landscapes of adult de novo acute myeloid leukemia. The New England journal of medicine. 2013;368(22):2059–74. 10.1056/NEJMoa1301689 23634996 PMC3767041

[40] Andrews S. FastQC: A quality control application for high throughput sequence data., Babraham Institute. Project page: http://www.bioinformatics.bbsrc.ac.uk/projects/fastqc. 2012.

[41] John JS. SeqPrep: Tool for stripping adaptors and/or merging paired reads with overlap into single reads. URL: https://githubcom/jstjohn/SeqPrep. 2011.

[42] Garrison E. vcflib: a C++ library for parsing and manipulating VCF files. URL: https://githubcom/ekg/vcflib. 2012.

[43] Tan A, Abecasis GR, Kang HM. Unified representation of genetic variants. Bioinformatics. 2015;31(13):2202–4. 10.1093/bioinformatics/btv112 25701572 PMC4481842

[44] Zook JM, Chapman B, Wang J, Mittelman D, Hofmann O, Hide W, et al Integrating human sequence data sets provides a resource of benchmark SNP and indel genotype calls. Nature biotechnology. 2014;32(3):246–51. 10.1038/nbt.2835 . 24531798

[45] Robinson JT, Thorvaldsdottir H, Winckler W, Guttman M, Lander ES, Getz G, et al Integrative genomics viewer. Nature biotechnology. 2011;29(1):24–6. 10.1038/nbt.1754 21221095 PMC3346182

[46] Thorvaldsdottir H, Robinson JT, Mesirov JP. Integrative Genomics Viewer (IGV): high-performance genomics data visualization and exploration. Briefings in bioinformatics. 2013;14(2):178–92. 10.1093/bib/bbs017 22517427 PMC3603213

