# Supplementary material for: ExScalibur: A High-Performance Cloud-Enabled Suite for Whole Exome Germline and Somatic Mutation Identification
Source: PLoS One. 2015 Aug 13;10(8):e0135800. doi: 10.1371/journal.pone.0135800 (PMC4535852; doi:10.1371/journal.pone.0135800)
Supplement: S1 File — Horizontal bars represent the progress of each module. Text on/next to each bar indicates sample/read group and software information. Runtime is shown as x-axis at the bottom of the panel. Task information and system settings not shown. (HTML) [file pone.0135800.s001.html]

## BigDataScript report: Run\_ExScaliburGMD.bds

|  |  |
| --- | --- |
| Script file | Run\_ExScaliburGMD.bds |
| Program ID | Run\_ExScaliburGMD.bds.20150326\_004411\_064 |
| Start time | 2015-03-26 00:44:11 |
| Run time | 00:52:58.462 |
| Tasks executed | 83 |
| Tasks failed | 0 |
| Tasks failed names |  |
| Arguments\* | [-aligners, bwamem, -callers, freebayes, -projdir, /data/rbao/BDS-ExScaliburGMD-032215/LCAexomeProj, -project, LCAexome, -samples, LCAdau, father, mother] |
| System\* | sge |
| Cpus\* | 1 |
| Exit value | 0 |

**\*** Values in global scope when program finished execution.

## Timeline
