# Supplementary figures and images for: ExScalibur: A High-Performance Cloud-Enabled Suite for Whole Exome Germline and Somatic Mutation Identification
Source: PLoS One. 2015 Aug 13;10(8):e0135800. doi: 10.1371/journal.pone.0135800 (PMC4535852; doi:10.1371/journal.pone.0135800)

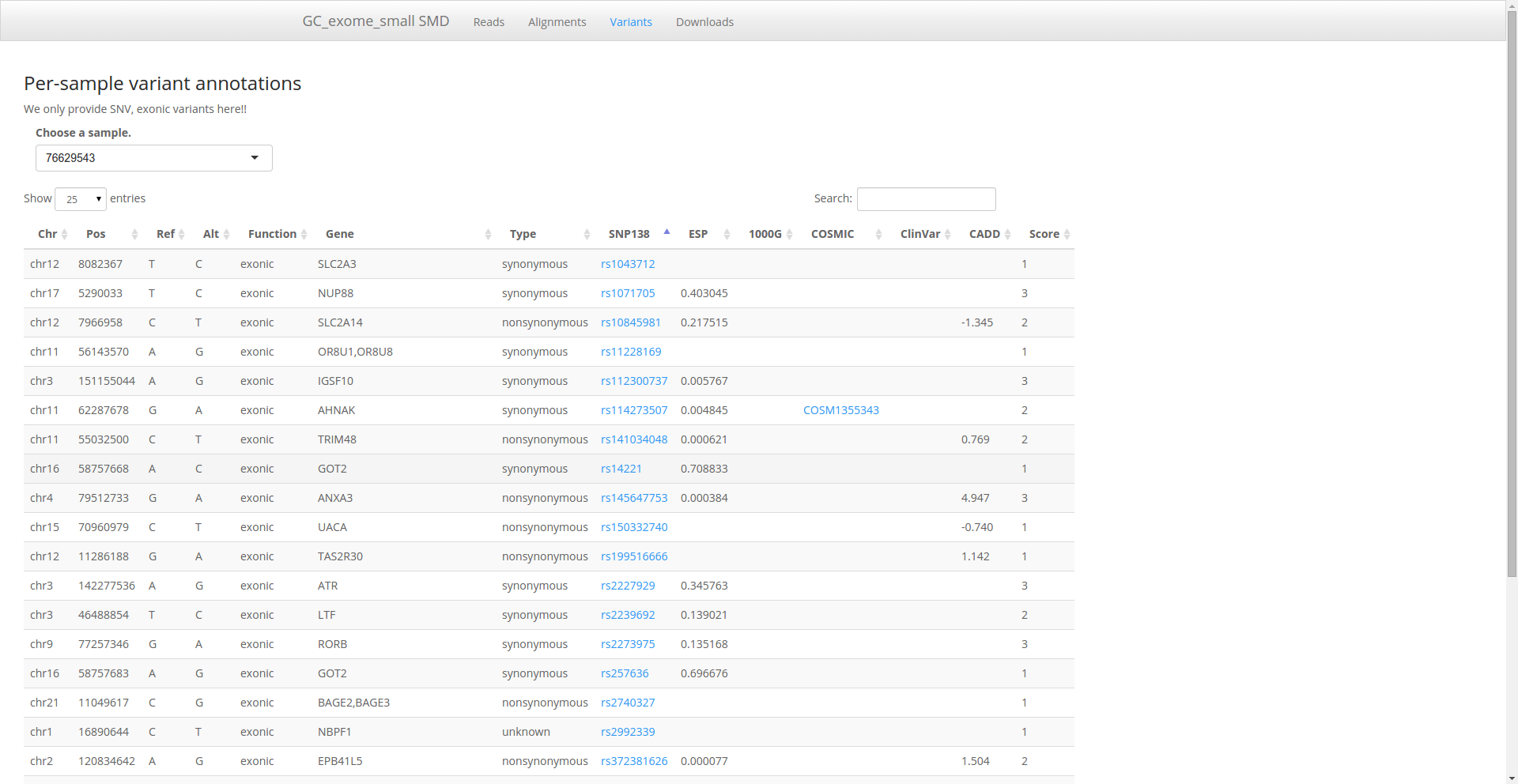

Supplement: S1 Fig — (TIF) [file pone.0135800.s002.tif]

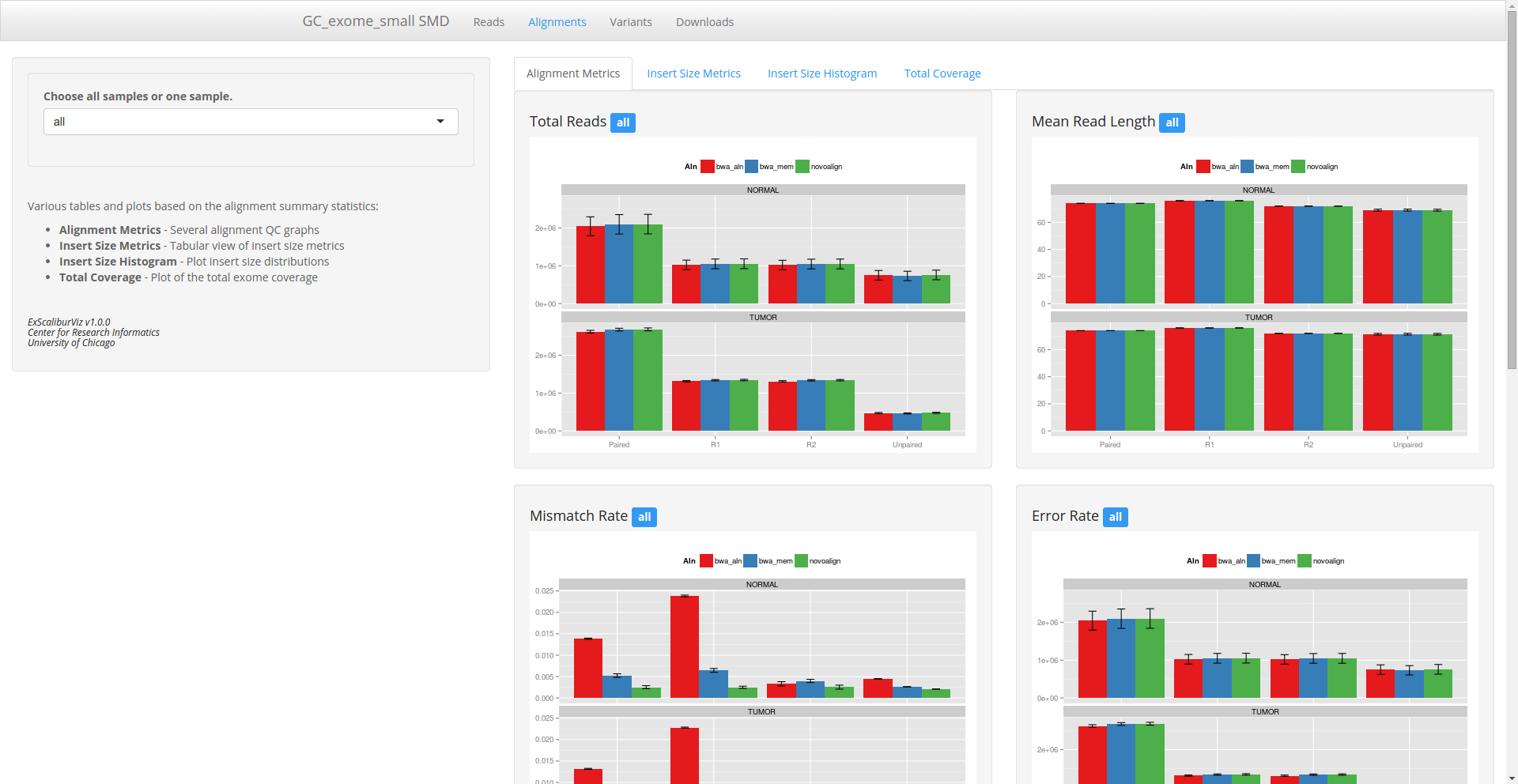

Supplement: S2 Fig — (TIF) [file pone.0135800.s003.tif]

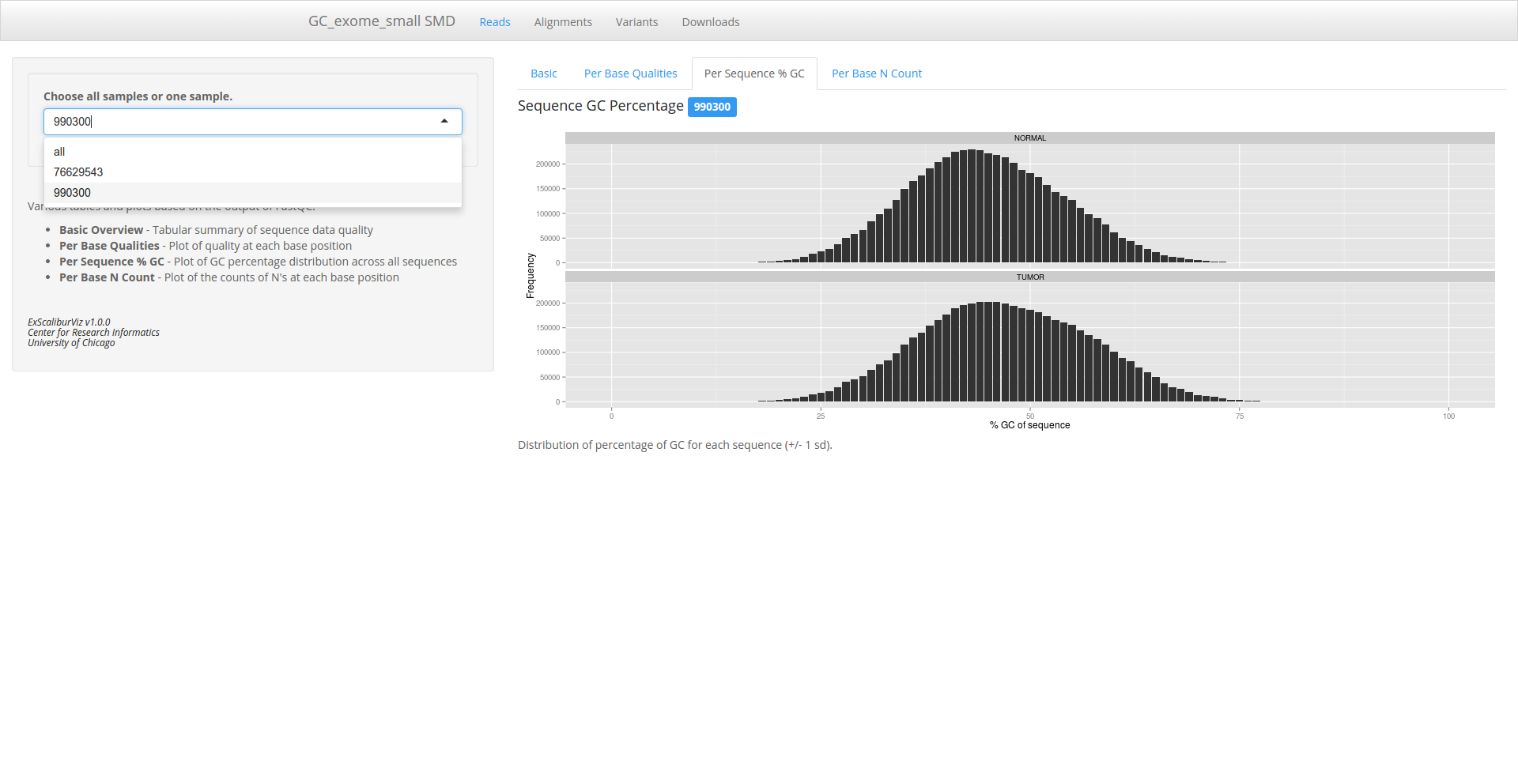

Supplement: S3 Fig — (TIF) [file pone.0135800.s004.tif]

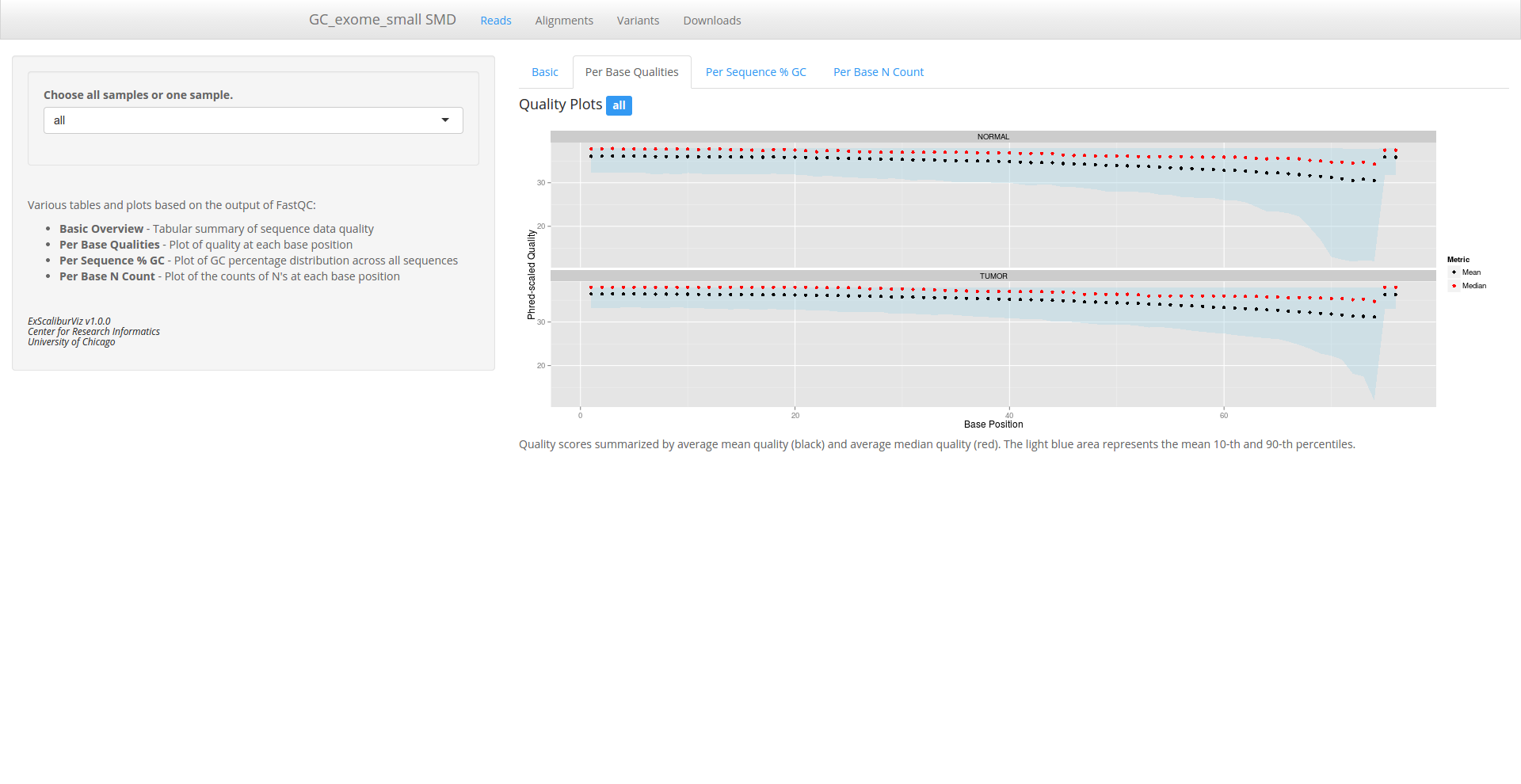

Supplement: S4 Fig — (TIF) [file pone.0135800.s005.tif]
